# Oncological Outcomes of Robotic-Assisted Surgery With High Dissection and Selective Ligation Technique for Sigmoid Colon and Rectal Cancer

**DOI:** 10.3389/fonc.2020.570376

**Published:** 2020-10-21

**Authors:** Tzu-Chieh Yin, Wei-Chih Su, Po-Jung Chen, Tsung-Kun Chang, Yen-Cheng Chen, Ching-Chun Li, Yi-Chien Hsieh, Hsiang-Lin Tsai, Ching-Wen Huang, Jaw-Yuan Wang

**Affiliations:** ^1^Division of General and Digestive Surgery, Department of Surgery, Kaohsiung Medical University Hospital, Kaohsiung Medical University, Kaohsiung, Taiwan; ^2^Department of Surgery, Kaohsiung Municipal Tatung Hospital, Kaohsiung Medical University, Kaohsiung, Taiwan; ^3^Division of Colorectal Surgery, Department of Surgery, Kaohsiung Medical University Hospital, Kaohsiung Medical University, Kaohsiung, Taiwan; ^4^Division of Colorectal Surgery, Department of Surgery, Kaohsiung Municipal Hsiaokang Hospital, Kaohsiung, Taiwan; ^5^Department of Surgery, Faculty of Medicine, College of Medicine, Kaohsiung Medical University Hospital, Kaohsiung Medical University, Kaohsiung, Taiwan; ^6^Graduate Institute of Medicine, College of Medicine, Kaohsiung Medical University, Kaohsiung, Taiwan; ^7^Graduate Institute of Clinical Medicine, College of Medicine, Kaohsiung Medical University, Kaohsiung, Taiwan; ^8^Center for Cancer Research, Kaohsiung Medical University, Kaohsiung, Taiwan

**Keywords:** selective ligation of IMA, sigmoid colon cancer, rectal cancer, robotic surgery, oncologic outcomes

## Abstract

**Background:** Curative resection of sigmoid colon and rectal cancer includes “high tie” of the inferior mesenteric artery (IMA). However, IMA ligation compromises blood flow to the anastomosis, which may increase the complication rate. We present preliminary experiences of operative and oncologic outcomes of patients with rectal or sigmoid colon cancer who underwent robotic surgery employing the high dissection and selective ligation technique.

**Methods:** Over May 2013 to April 2017, 113 stage I–III rectal or sigmoid colon cancer patients underwent robotic surgery with the single-docking technique at one institution. We performed D3 lymph node dissection and low-tie ligation of the IMA (i.e., high dissection and selective ligation technique). Clinicopathological features, perioperative parameters, and postoperative outcomes were retrospectively analyzed. Overall survival (OS) and disease-free survival (DFS) were calculated using the Kaplan–Meier method.

**Results:** Sphincter preservation rate was 96.3% in rectal cancer patients. Median number of harvested lymph nodes was 12. Apical nodes were pathologically harvested in 84 (82.4%) patients. R0 resection was performed in 108 (95.6%) patients. Overall complication rate was 17.7%; but most complications were mild and the patients recovered uneventfully. Estimated 5-year OS was 86.1% and 3-year DFS was 79.6% after median follow-up periods of 49.1 months (range, 5.3–85.3).

**Conclusions:** High dissection of the IMA and selective ligation of the major feeding vessel to the sigmoid colon or rectum can be safely performed using da Vinci Surgical System,yielding favorable clinical, and oncologic outcomes in rectal or sigmoid colon cancer treatment.

## Introduction

Total mesorectal excision (TME) surgery, notably improves the clinical outcomes, has served as the essential procedure for patients with rectal cancer since it was first described by Heald and Ryall in 1982 (1). MacFarlane et al. (2) reported a 5-year local recurrence rate of 5% in patients who underwent TME surgery alone. Moreover, in patients with locally advanced rectal cancer (LARC), local recurrence rate is substantially reduced by preoperative concurrent chemoradiotherapy (CCRT). A German study reported that the 5-year cumulative incidence of local relapse was 6% in patients who received preoperative CCRT (3, 4). Similar results were reported in other studies (5–7), and preoperative CCRT with subsequent surgical intervention has been highly recommended as the treatment for patients with of LARC (8).

Laparoscopic rectal surgery with TME is highly technically dependent and requires skilled surgeons experienced in minimal invasive surgery (9, 10). The robotic system (da Vinci Surgical System, Intuitive Surgical, Inc., Sunnyvale, CA, USA) has several strengths including high-definition three-dimensional magnified vision, the surgeon-controlled camera platform, the meticulous articulatory instruments, and steady traction provided by the robotic arm. Operation in the confined and narrow space such as pelvic cavity can be performed more easily and precisely. Robotic colon surgery, which was first performed in 2002 (11), is expected to address the disadvantages of conventional laparoscopic colorectal surgery. Compared with laparoscopic surgery, robotics rectal cancer surgery is more favorable in regarding to clinical and short-term oncological outcomes and is suggested in several studies (12–15). Even in patients of LARC or mid to low rectal cancer received CCRT, robotic rectal surgery is still associated with at least comparable short-term surgical outcomes (16–18).

Conventionally, high ligation of the inferior mesenteric artery (IMA) is one of the important surgical steps in rectal or sigmoid colon cancer surgery. However, this technique may reduce blood flow in the bowel and increase the risk of ischemia and anastomosis leakage (19). In a study of patients with rectal cancer following preoperative CCRT, Huang et al. (20) observed that robotic surgery with the technique of high dissection and low ligation resulted in a sufficient number of harvested lymph nodes and a low rate of anastomotic leakage. Because the high dissection and selective ligation of IMA branches theoretically preserves more blood supply to the anastomosis, we intend to apply this technique by using the robotic system in patients with rectal cancer or sigmoid colon cancer and evaluate their perioperative surgical, pathologic, and oncologic outcomes.

## Materials and Methods

### Patients

This is a single-institution retrospective study. Patients with stage I–III rectal or sigmoid colon cancer underwent robotic surgery with the single-docking technique were enrolled into this study. This study was approved by the institutional review board of our hospital. Informed consent was obtained from each patient before the robotic surgery was performed. Over May 2013 to April 2017, 113 consecutive patients were included in the study and analyzed. These patients routinely underwent diagnostic colonoscopy and abdominal and pelvic computed tomography (CT) or magnetic resonance imaging for preoperative staging. Patients with T3/T4 or N+ rectal cancer received preoperative CCRT. A biweekly 5-fluorouracil, leucovorin, and oxaliplatin (FOLFOX) chemotherapy regimen or a fluoropyrimidine-based regimen was used for CCRT in patients with LARC. Long-course radiotherapy was concurrently administered according to our protocol (20, 21). The total radiation dose was delivered in a range of 45–50.4 Gy using a daily fraction of 1.8–2.0 Gy. All patients received external-beam radiotherapy with either three-dimensional conformal or intensity-modulated radiation therapy. Most of the robotic-assisted surgery with the single-docking technique were scheduled ~8–12 weeks after completion of radiotherapy (22).

Clinicopathological features and perioperative parameters or outcomes, including age, sex, histological type, TNM classification, time interval between completion of preoperative radiotherapy and robotic surgery, tumor location (distance from the anal verge), American Society of Anesthesiologists classification, and body mass index (BMI), were collected and evaluated. The TNM classification was defined according to the American Joint Commission on Cancer (AJCC)/International Union Against Cancer (UICC) criteria (23). The tumor regression grade (TRG) was assessed according to the AJCC system (24). Perioperative outcomes, including surgical procedures, time consumed in docking, console and operation, estimated blood loss, time of the first flatus passage, time of resuming soft diet, length of hospital stay, and pain score (visual analog scale) at the first postoperative day, were collected and evaluated.

Patients were regularly followed up after operation, which included the collection of clinical outcome and survival status data. History-taking and physical examinations were performed every 3 months postoperatively. Serum carcinoembryonic antigen levels were measured every 2–3 months postoperatively. Colonoscopy was carried out around 1 year after surgery. A repeat colonoscopy was generally recommended at 3 years. In patients with stage II–III disease, abdominal, and pelvic CT scans were executed annually for 3 consecutive years postoperatively.

### Surgical Procedures

**Figure 1:**
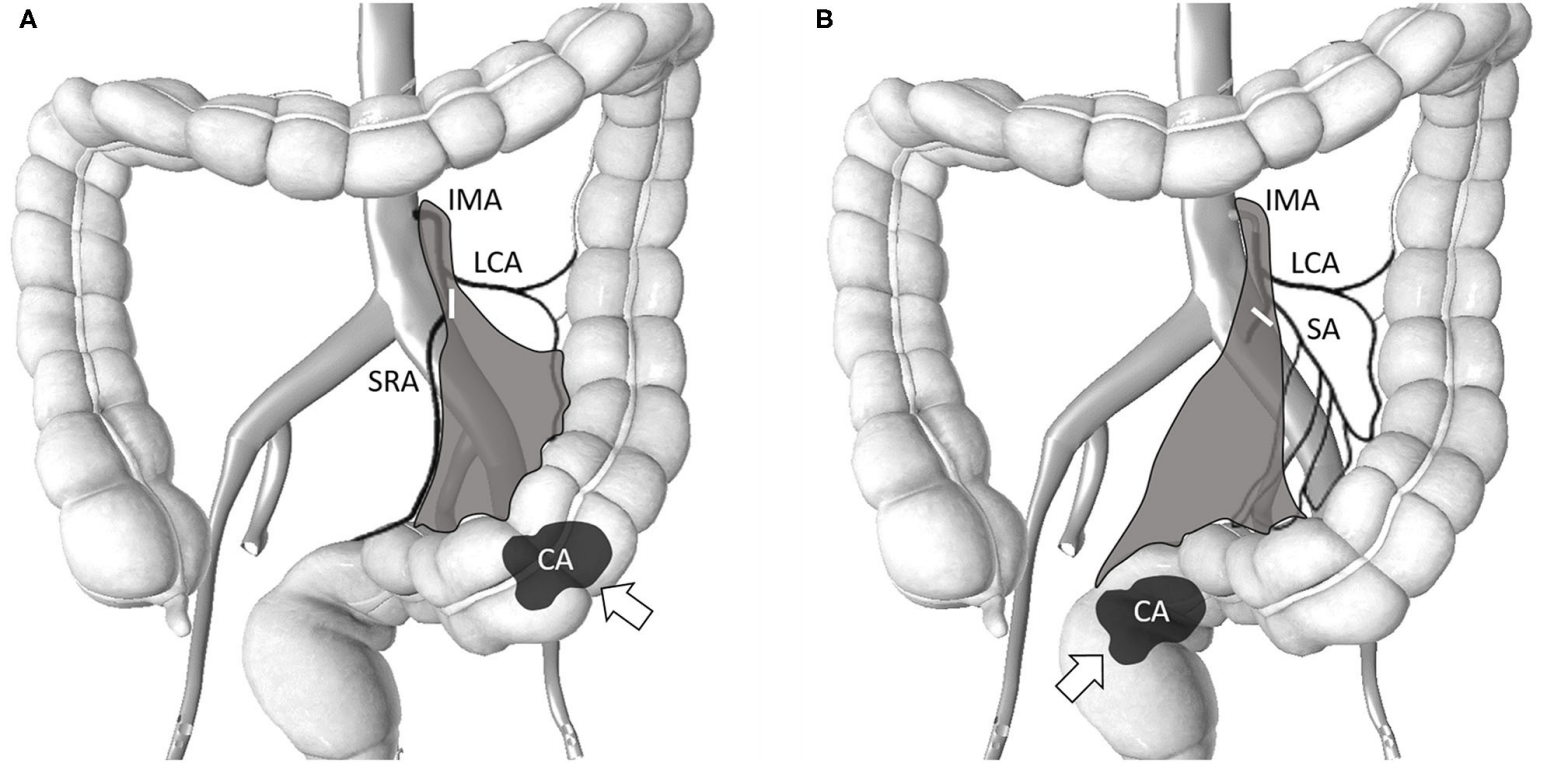
Schematic of the area (gray-color) of lymph node clearance in high dissection and selective ligation. The origin of the IMA from the abdominal aorta and the junction of the IMA and LCA was explored. Lymphoadipose tissues around this area were skeletonized and stripped to facilitate complete D3 lymph node dissection. The short white bar denotes the level of the major feeding vessel ligated and transected. **(A)** Selective ligation of the SA with preservation of the LCA for sigmoid colon cancer. The SRA was also preserved during AR when the tumor was located in the proximal sigmoid colon; **(B)** Selective ligation of the SRA with preservation of the LCA for rectal cancer. The SA was also preserved in case of rectal cancer with redundant sigmoid colon. IMA, inferior mesenteric artery; LCA, left colic artery; SA, sigmoid artery; SRA, superior rectal artery; AR, anterior resection; CA and arrow denotes the tumor location.

The da Vinci Si surgical system was docked over the left flank of the patient. We used medial to lateral fashion to ligate and divide the branches of inferior mesenteric artery and vein. First, a peritoneal incision at the level of the sacral promontory was performed. The dissection was then extended cranially and caudally. We fully explored the IMA from the abdominal aorta to the converging of the left colic artery (LCA) and IMA. Lymphoadipose tissues here were skeletonized and stripped easily with the use of the da Vinci Surgical System to complete the lymph nodes dissection over the root of IMA. We performed dissection of the D3 lymph node (from the level of the IMA entering the abdominal aorta to the branching of the LCA) and low-tie ligation of the IMA with preservation of the LCA in all patients; this technique also known as the high dissection and low ligation technique (20). Transection was executed below the junction of IMA and LCA. The superior rectal artery (SRA) was also preserved during anterior resection (AR) if the tumor was located in the proximal sigmoid colon. In addition, the sigmoid artery was preserved in case of rectal cancer with redundant sigmoid colon (Figure 1). The inferior mesenteric vein (IMV) was identified but was not transected immediately. The IMV was ligated and divided only if excess tension was noted during the colonic anastomosis. The splenic flexure was not routinely mobilized if its mobilization was also dependent on the tension of the anastomosis. Totally robotic-assisted TME with the single-docking technique was performed on patients of rectal cancer as described previously (22).

After complete mobilization of the sigmoid colon, mesocolon, entire rectum and TME, AR using the hand-sewn or double-stapled technique, low anterior resection (LAR) using the double-stapled technique, intersphincteric resection (ISR) with coloanal anastomosis as well as protective diverting colostomy, or abdominoperineal resection (APR) was performed accordingly. A specimen was then extracted and resected transanally after ISR. Finally, a conventional laparoscope was used to meticulously inspect for the abdominal and pelvic cavity, and a drain tube was placed in the cul-de-sac.

### Statistical Analysis

All data were statistically analyzed using JMP for Windows (version 13.0; SAS Institute, Cary, NC, USA). Continuous variables are presented as median and their quartiles, and dichotomous variables are denoted by numbers and percentages. A *t* test was used to analyze continuous variables, and the Chi-square test was used for univariate statistical analysis. All patients were followed up until their death, final follow-up, or May 31, 2020. The survival plot was calculated using the Kaplan–Meier method.

## Results

### Patients Characteristics and Perioperative Outcomes

The baseline characteristics and perioperative outcomes of 113 patients with rectal or sigmoid colon cancer who underwent robotic-assisted surgery using the high dissection and selective ligation technique are summarized in Table 1. The median age and BMI of the patients was 62 (range, 28–88) years and 24.0 (IQR, 22.1–26.2) kg/m^2^, respectively. Of the 113 patients, 82 (50.5%), 22 (31.6%), and 9 (17.9%) had rectal, rectosigmoid, and sigmoid colon cancers, respectively. The median distance of the tumor from the anal verge was 5 (IQR, 3–7) cm in patients with rectal cancer. The most frequent surgical procedure was LAR (66, 58.4%), followed by ISR with coloanal anastomosis (35, 31.0%), and APR (3, 2.7%).

**Table 1:** Baseline characteristics and perioperative outcomes of 113 patients with rectal or sigmoid colon cancer who underwent totally robotic-assisted TME with the high dissection and selective ligation technique.

**Characteristics**	
Age (years, median) (range)	62 (28–88)
**Sex**	
Male	69 (61.1%)
Female	44 (38.9%)
BMI (IQR)	24.0 (22.1–26.2)
Distance from anal verge, rectum only (cm, median) (IQR)	5 (3–7)
**Pre-operative CCRT**	
Yes	79 (69.9%)
No	34 (30.1%)
**Pre-operative chemotherapy regimen**	
FOLFOX	58 (73.4%)
Fluoropyrimidine-based	21 (26.6%)
Time interval (days, median) (range)	82 (41–203)
**ASA classification**	
II	64 (56.6%)
III	49 (43.4%)
**Tumor location**	
Sigmoid colon	9 (17.9%)
Recotosigmoid colon	22 (31.6%)
Rectum	82 (50.5%)
**Procedure**	
AR	9 (8.0%)
LAR	66 (58.4%)
ISR	35 (31.0%)
APR	3 (2.7%)
Protective colostomy (except APR)	44 (40%)
Docking time (min, median) (IQR)	5 (4–6)
Console time (min, median) (IQR)	205 (168–244)
Operation time (min, median) (IQR)	320 (280–436)
Estimate blood loss (mL, median) (IQR)	80 (50–145)
Time to flatus passage (day, median) (IQR)	2 (1, 2)
Time to resume soft diet (day, median) (IQR)	4 (3,4)
Postoperative LOS (day, median) (range)	6 (5–32)
POD1 VAS pain score (median) (IQR)	3 (3–4)

*BMI, body mass index; TME, total mesorectal excision; CCRT, concurrent chemoradiotherapy; ASA, American Society of Anesthesiologists; AR, anterior resection; LAR, low anterior resection; ISR, intersphincteric resection; APR, abdominoperineal resection; LOS, length of stay, POD1: postoperative day 1*.

Finally, 44 patients (40%) had protective diverting colostomy after surgery, including 35 patients and 9 patients who underwent ISR and LAR, respectively. Sphincter preservation rate was 79/82 (96.3%) in patients with rectal cancer. The median estimated blood loss, including tissue fluid after CCRT, was 80 mL. The median time of the first flatus passage and resuming soft diet was 2 and 4 days postoperative, respectively, and the median length of postoperative hospital stay was 6 (range, 5–32) days.

### Postoperative Complications

Postoperative complications are summarized in Table 2. Postoperative complications were observed in 13 patients with 20 episodes (17.7%). One (0.9%) patient developed intraabdominal bleeding and unstable hemodynamic condition postoperatively; a laparotomy was subsequently performed, during which bleeding from the mesocolon was noted. Anastomosis leakage occurred in 4 (3.5%) patients who underwent LAR with the double-stapled technique. 3 of them were male, 3 had preoperative CCRT and protective diverting stoma was not created simultaneously in these 4 patients. Loop colostomy of the transverse colon was subsequently performed. Five (4.4%) patients developed stenosis of coloanal anastomosis and underwent colonoscopic balloon dilation. Urethral injury during ISR was noted in one patient (0.9%). All postoperative ileus, pulmonary complications, and urinary tract infection were of grades I–II according to the Clavien–Dindo classification, and the patients recovered after conservative treatment. Furthermore, no 30-day hospital mortality occurred.

**Table 2:** Postoperative complications of 113 patients with rectal or sigmoid colon cancer who underwent totally robotic-assisted TME with the high dissection and selective ligation technique.

**Complications**	**Number (%)**	**Management**
Postoperative bleeding	1 (0.9%)	Laparotomy
Anastomosis leakage	4 (3.5%)	Loop transverse colostomy
Intraabdominal infection/abscess	2 (1.8%)	1 CT guide drainage
		1 Conservative treatment
Coloanal anastomosis stenosis	5 (4.4%)	Colonoscopic dilation
Urethral injury	1 (0.9%)	Conservative treatment
Postoperative Ileus	3 (2.7%)	Conservative treatment
Pulmonary complication	3 (2.7%)	Conservative treatment
Urinary tract infection	1 (0.9%)	Conservative treatment
Total	20 (17.7%)	

*TME, total mesorectal excision*.

### Clinicopathological Features and Oncological Outcomes

The pathological characteristics and oncological outcomes of all 113 patients are summarized in Table 3. Most of the patients had LARCs in preoperative clinical staging including T3 in 73 (64.6%) patients, T4 in 15 (12.3%) patients, or N+ in 61 (54.0%) patients. Therefore, preoperative CCRT was carried out in 79 (69.9%) patients, including FOLFOX regimen in 58 (73.4%) patients and fluoropyrimidine-based regimen in 21 (26.6%) patients. The median number of harvested lymph nodes and apical lymph nodes was 12 (IQR, 8–17) and 2 (IQR, 1–4), respectively. However, metastatic apical lymph node was found in only 3 (3.6%) patients. The median distance of the circumferential resection margin (CRM) and the distal resection margin (DRM) was 1.0 and 2.0 cm, respectively. CRM and DRM involvement were noted in 4 (3.5%) and 1 (0.9%) patients, respectively. R0 resection (microscopic tumor clearance) was accomplished in 108 (95.6%) patients. Pathologic complete response (pCR) of the primary tumor was observed in 28 (36.8%) of the 79 patients who received preoperative CCRT. In total, 28 (36.8%), 32 (42.1%), 10 (13.2%), and 6 (7.9%) patients revealed complete (TRG 0), moderate (TRG 1), minimal (TRG 2), and poor (TRG 3) response, respectively. The median time interval between radiotherapy completion and robotic surgery was 82 (range, 41–203) days.

**Table 3:** Pathological characteristics and oncological outcomes of 113 patients with rectal or sigmoid colon cancer who underwent totally robotic-assisted TME with the high dissection and selective ligation technique.

**Clinical staging**	
**Tumor depth**	
T1	2 (1.8%)
T2	23 (20.4%)
T3	73 (64.6%)
T4	15 (12.3%)
**Lymph node metastasis**	
N0	52 (46.0%)
N1	38 (33.6%)
N2	23 (20.4%)
**AJCC Stage**	
I	22 (19.5%)
II	30 (26.5%)
III	61 (54.0%)
**Pathological outcomes**	
**Histology**	
WD	15 (13.4%)
MD	94 (83.9%)
PD	3 (2.7%)
**Tumor size**	
<5 cm	102 (90.3%)
≧5 cm	11 (9.7%)
Tumor size (cm, median) (IQR)	2.5 (1.2–3.2)
**Tumor depth**	
T0	31 (27.4%)
Tis	1 (0.9%)
T1	21 (18.6%)
T2	25 (22.1%)
T3	32 (28.3%)
T4	3 (2.7%)
**Lymph node metastasis**	
N0	87 (77.0%)
N1	19 (16.8%)
N2	7 (6.2%)
**AJCC stage**	
0	30 (26.5%)
I	38 (33.6%)
II	19 (16.8%)
III	26 (23.0%)
**Pathological response grade (in 79 CCRT)**	
0	28 (36.8%)
1	32 (42.1%)
2	10 (13.2%)
3	6 (7.9%)
**Lymph node harvested (median) (IQR)**	
With CCRT	10 (8–14)
Without CCRT	17 (12–21)
Apical node harvested	84 (82.4%)
Apical node harvested (median) (IQR)	2 (1–4)
Positive apical node	3 (3.6%)
Length of bowel resected (median) (IQR)	10.5 (9.0–12.5)
Distance of distal resection margin, rectum only (cm, median) (IQR)	2.0 (1.1–3.0)
Distance of circumferential resection margin, rectum only (cm, median) (IQR)	1.0 (0.4–1.5)
**Distal resection margin**	
Free	112 (99.1%)
Positive	1 (0.9%)
**Circumferential resection margin**	
Free	109 (96.5%)
Positive	4 (3.5%)
**Resection degree**	
R0	108 (95.6%)
R1	5 (4.4%)
**Oncological outcomes**	
Follow up periods (month, median) (range)	49.1 (5.3–85.3)
**R0 resection**	
Locoregional recurrence	8 (7.4%)
Distant metastasis	12 (11.1%)
Liver	2 (1.9%)
Lung	6 (5.6%)
Chest wall + adrenal gland	1 (0.9%)
Chest wall + bone	1 (0.9%)
Non-regional LN	2 (1.9%)
**R1 resection**	
Locoregional recurrence	3 (60%)
Distant metastasis	4 (80%)
Liver	2 (40%)
Lung	1 (20%)
Peritoneal carcinomatosis	1 (20%)

*TME, total mesorectal excision; AJCC, American Joint Commission on Cancer; WD, well-differentiated; MD, moderately-differentiated; PD, poorly-differentiated; CCRT, concurrent chemoradiotherapy*.

**Figure 2:**
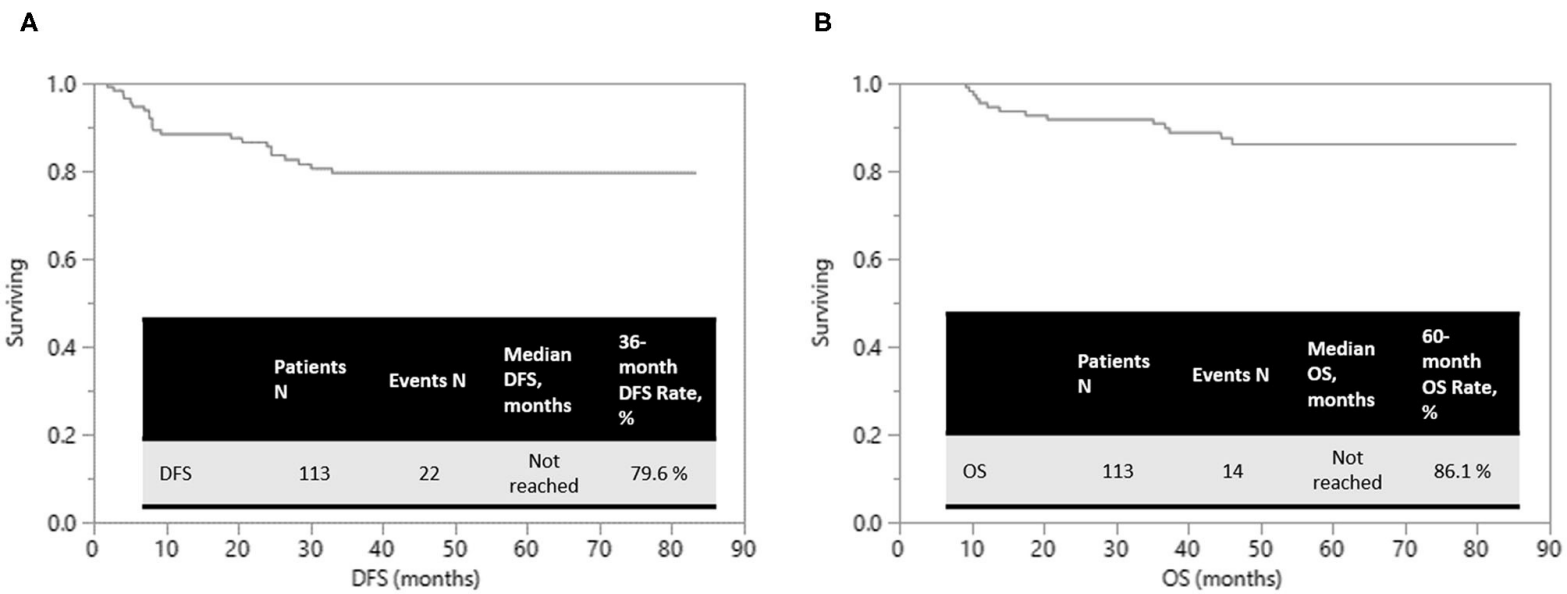
The Kaplan–Meier survival curves. **(A)** Overall survival. **(B)** Disease-free survival.

Of 108 patients who underwent R0 resection, local recurrence, and distant metastases happened in 8 (7.4%) and 12 (11.1%) patients, respectively. At a median follow-up duration of 49.1 (range, 5.3–85.3) months, the estimated 5-year overall survival (OS) was 86.1% and 3-year disease-free survival (DFS) was 79.6%, respectively (Figure 2).

## Discussion

The completeness of TME is the indication of the qualification of rectal cancer surgery. CRM involvement and DRM distance constitute the two crucial parameters of TME completeness. Moreover, CRM involvement may be a prognostic factor for local recurrence and survival (25–28). In this study, the rate of CRM and DRM involvement were 3.5 and 0.9%, with a median distance of 1.0 and 2.0cm, respectively. These were comparable to that reported in a previous study (0–16.1% of CRM involvement and 1.5–3.9 cm of DRM distance) (22). The odds of achieving R0 resection in rectal cancer patients increased with the use of robotic-assisted surgery (29). The robotic platform also reduced the difficulty of accomplishing R0 resection in cancer requiring extensive resection (30). In current study, R0 resection for all cases was performed in 108 of 113 patients (95.6%). Of the 108 patients who underwent R0 resection, local recurrence and distant metastasis occurred in 8 (7.4%) and 12 (11.1%), respectively.

Although 69 of our patients (61.1%) were men and 75 (66.4%) of our patients had middle to low rectal cancers (tumor located at ≤ 10 cm from the anal verge) with a median distance of 5 cm from the anal verge, we did not routinely mobilize the splenic flexure in most of them. Precise dissection during the TME procedure could still be executed when using our single-docking technique. Splenic flexure was mobilized only as required to achieve a tension-free anastomosis (31, 32). However, a comparable DRM distance and favorable negative rates of CRM were reached. The introduction of extended oxaliplatin-based chemotherapy and a longer interval between radiotherapy completion and robotic surgery might have contributed to the success (8, 33). The sphincter preservation rate in patients with rectal cancer in our study was 96.3%, comparable to that reported by Kim et al. (18) and Saklani et al. (16). The anastomosis leakage rate (3.5%) in our study was lower than that reported in literature (7.1–16%) (34–37) even though protective diverting colostomy was performed in only 44 (55.7%) of 79 patients who underwent sphincter preservation surgery. Cautiously protective diverting stoma performing in high risk patients may further decrease the leakage rate.

The status of lymph node metastasis is a key factor for predicting the prognoses of patients with colorectal cancer (CRC). The AJCC/UICC recommends that at least 12 lymph nodes should be examined for each surgical specimen of CRC (23). However, the guidelines were mainly based on colon cancer studies. Patients with rectal cancer and older patients with distally located early colon cancer were less likely to meet the recommended lymph node yield (i.e., 12) (38). Moreover, the number of harvested lymph nodes essentially decreased after preoperative CCRT (median, 4–14) (39). In the present study, the median number of harvested lymph nodes after neoadjuvant CCRT was 10 (range, 8–14), which is consistent with previous results (39).

IMA ligation level remains a controversial topic. To obtain sufficient lymph nodes is the main purpose of high ligation of the IMA for better survival and adequate staging. However, this technique may result in bowel ischemia and subsequent anastomosis leakage and/or stenosis due to reduced blood flow in the colon. Recently, some surgeons suggest the technique of low ligation of the IMA with preservation of the LCA (19, 34, 40). In addition, the metastatic rate of lymph nodes around the IMA was reported to be 0.3–8.6% in patients with rectal cancer (41). In the present study, we performed high dissection of the IMA and selective ligation of the major feeding vessel to the sigmoid colon or rectum. Apical lymph nodes were identified in 84 (82.4%) of 102 patients, and the median number of harvested apical lymph nodes was 2 (range, 1–4). However, positive apical lymph node metastasis was observed in only three patients (3.6%). Therefore, we obtained results comparable to those in the literature, including sufficient harvesting rate and number of harvested apical lymph nodes, with this technique (35, 36, 42). The routine ligation of IMA might be re-considered if it remained as a standard operative procedure.

Studies regarding high dissection and selective ligation are briefly summarized in Table 4. Our technique was similar to that described in the literature, including low ligation of the IMA, IMA preservation, LCA preservation, low tie with lymph node dissection, and low ligation with preservation of the LCA (35, 37, 42–44). This technique was mostly used in AR and LAR for stage I–III sigmoid colon or rectal cancer during lymph node dissection at the root of the IMA. Oncologic outcome and complication rate were the most pertinent points in these studies. OS (42, 43) and DFS (42–44) were not inferior to those in patients who received standard high ligation technique procedures even in N+ or ≥T3 disease (42). In some cases with proximal sigmoid colon cancer, both the LCA and SRA were preserved (44) and the DFS was unaltered.

**Table 4:** Studies regarding the high dissection and selective ligation technique published since 2013.

**Study**	**Country (year)**	**Patient number**	**Tumor location**	**Description of the technique**	**Comparison**
(37)	Japan (2013)	155	Middle and low rectum	LCA preserving	Operative outcome, complications, OS RFS
(42)	Japan (2014)	120	Sigmoid colon and RS colon, T3	Low ligations with preservation of the LCA	Operative outcome, OS, DFS
(35)	Japan (2015)	49	Rectum	Low-ligation of IMA	Defecatory function, QoL, leakage rate, LN harvested
(43)	Japan (2016)	147	Sigmoid colon and rectum	Low tie with lymph node dissection	OS, DFS, complications
(44)	Japan (2018)	142	Sigmoid colon and upper rectum	IMA preservation	Operative outcome, LN harvested, OS, DFS
Present study	Taiwan (2020)	113	Sigmoid colon and rectum	High dissection and selective ligation technique	Operative outcomes, complications, pathologic outcomes, OS, DFS

*LCA, left colic artery; OS, overall survival; DFS, disease free survival; RS, rectosigmoid; IMA, inferior mesenteric artery; QoL, quality of life; LN, lymph node*.

Anastomotic leakage rate was as low as 3.5% in the present study with the robotic-assisted high dissection and selective ligation technique. Nevertheless, complication and anastomotic leakage rates revealed equivalent outcomes for the high-tie and low-tie techniques for sigmoid colon and rectal cancer surgery in the study of Yasuda et al. (43). Favorable anastomotic leakage rate (7.4 vs. 13.2%; hazard ratio 0.269 (0.131–0.550); *P* < 0.001) was observed in LCA preservation during laparoscopic-assisted sphincter-preserving rectal surgery (37). A low complication rate, including only 6% anastomotic leakage rate, was also achieved in a study by Liang et al. (34) by using the robotic approach to perform D3 lymph node dissection over the IMA with preservation of the LCA for the treatment of distal rectal cancer.

This study has some limitations. First, this was a single-institution retrospective study including only 113 patients. Second, the median interval of follow-up was 49.1 months; however, the 3-year local control rate (89.4%) and the 3-year distant metastasis control rate (84.7%) were comparable with those reported in related studies.

## Conclusions

Robotic-assisted high dissection and selective ligation of the IMA yields low postoperative complication rates, enables the harvesting of sufficient lymph nodes, and provides equivalent oncologic outcome compared with the conventional high ligation technique. R0 resection remains one of the most critical elements for locoregional control and the reduction of distant metastasis. This effective and safe surgical technique, with favorable oncological outcomes, is suggested for the potential treatment option for patients with stage I–III rectal cancer or sigmoid cancer.

## Data Availability Statement

The raw data supporting the conclusions of this article will be made available by the authors, without undue reservation.

## Ethics Statement

The studies involving human participants were reviewed and approved by Institutional Review Board of Kaohsiung Medical University Hospital. The patients/participants provided their written informed consent to participate in this study.

## Author Contributions

T-CY, being the first author of this manuscript, designed this study, analyzed the data, and wrote the manuscript. W-CS, P-JC, T-KC, Y-CC, C-CL, Y-CH, H-LT, and C-WH made substantial contributions in terms of the data acquisition, interpretation and statistical analyses, in addition to assisting with the manuscript preparation. J-YW, being the corresponding author for this manuscript, also participated in the study design and coordination, in addition to making critical revisions to the manuscript. All authors have reviewed and approved submission of the final version of the manuscript.

## Conflict of Interest

The authors declare that the research was conducted in the absence of any commercial or financial relationships that could be construed as a potential conflict of interest.
